# Physical Modeling of Gate-Controlled Schottky Barrier Lowering of Metal-Graphene Contacts in Top-Gated Graphene Field-Effect Transistors

**DOI:** 10.1038/srep18307

**Published:** 2015-12-17

**Authors:** Ling-Feng Mao, Huansheng Ning, Zong-Liang Huo, Jin-Yan Wang

**Affiliations:** 1 School of Computer & Communication Engineering, University of Science & Technology Beijing, 30 Xueyuan Road, Haidian District Beijing 100083, P. R. China; 2 Institute of Microelectronics, Chinese Academy Science 100029, Beijing, P. R. China; 3 Institute of Microelectronics, Peking University, 100871, Beijing, P. R. China

## Abstract

A new physical model of the gate controlled Schottky barrier height (SBH) lowering in top-gated graphene field-effect transistors (GFETs) under saturation bias condition is proposed based on the energy conservation equation with the balance assumption. The theoretical prediction of the SBH lowering agrees well with the experimental data reported in literatures. The reduction of the SBH increases with the increasing of gate voltage and relative dielectric constant of the gate oxide, while it decreases with the increasing of oxide thickness, channel length and acceptor density. The magnitude of the reduction is slightly enhanced under high drain voltage. Moreover, it is found that the gate oxide materials with large relative dielectric constant (>20) have a significant effect on the gate controlled SBH lowering, implying that the energy relaxation of channel electrons should be taken into account for modeling SBH in GFETs.

Zero band gap and linear energy-momentum relationship are the characteristic properties of the graphene, owing to its two-dimensional nature. Therefore the behavior of carriers in graphene is similar to massless Dirac fermions with speed of 10^6^ m s^−1^ 1. The effective electron mass in monolayer graphene has been obtained from experiments as 0.012m_0_ (m_0_ is the free electron mass)^2^. Graphene exhibits a high carrier mobility in the order of 20 000–200 000 cm^2^ V^−1^ s^−1^ at room temperature^3^, 16 000–42 000 cm^2^ V^−1^ s^−1^ ^4^, 10 000–15 000 cm^2^ V^−1^ s^−1^ for exfoliated graphene on SiO_2_ substrate, and over 100 000 cm^2^ V^−1^ s^−1^ for suspended samples^5^. This makes graphene as a promising candidate in ultra-fast electronic devices. A Schottky barrier is formed at the interface of the doped graphene-semiconductor junction^6^,^7^. In comparison with Si-based Schottky junction, experimental results showed that the SBH of grapheme transistor is strongly dependent on the gate voltage. It was observed that effective SBH decreases from 0.45  to 0.25 eV when the gate voltage increases from 0 to 5 V^7^. However, the explanation of the strong dependence of effective SBH on gate voltage is still not unclear. Yang *et al.*^7^ thought that the SBH could be tuned by adjusting the work function of graphene due to the absence of Fermi-level at the interface. Kim *et al.*^8^ believed that the image force also can lower SBH, which will result in some unreasonable conclusions shown in the following content. Chang *et al.*^25^ experimentally reported that the SBH lowering is caused by hot electrons for Schottky barrier nanowire charge-trapping silicon–oxide–nitride–oxide–silicon devices. It implies that the physical origin of the SBH lowering in field-effect transistors caused by hot electrons should carefully be considered. In this paper, the effect of hot-electron is firstly introduced to successfully account for SBH lowering of GFETs.

Hot-electron phenomena are important in all semiconductor devices, which are governed by inelastic interactions between carriers and phonons. Elastic and inelastic collisions are the dominant mechanisms for momentum and energy relaxations, respectively. Hot-electron phenomena in semiconductor devices can be analyzed by using the continuity equation, momentum conservation equation, and energy conservation equation^9^. For transistors with the gate length less than micrometer, the non-equilibrium nature of electrons and phonons must be considered^10^. The electron energy (electron temperature) can be much higher than lattice energy (lattice temperature) in semiconductor devices^9^,^10^,^11^, while the energy difference between electrons and lattices is governed by the energy relaxation (ER) time^12^. Experimental results showed that the ER time in a graphene device is about 1 ps, and the electron gas temperature varies from 400 K to 700 K when the lattice temperature is 300 K in single-wall carbon nano-tubes^13^. Moreover, a larger difference between the electron temperature and the lattice temperature in graphene could be found^14^.

## Results

### Theory

The aim of this study is to provide an explanation for gate-controlled Schottky barrier of a metal-graphene transistor on a silicon substrate reported in ref. 7. We thought that this modeling of the Schottky barrier height lowering effects could be used in any metal-semiconductor contact.

**Figure 1:**
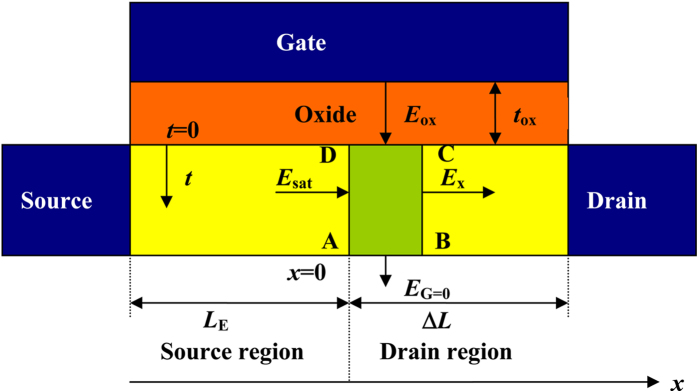
Schematic cross section of a top-gated graphene transistor.

The device architecture of a top-gated graphene field-effect transistor can be simplified as the structure shown in Fig. 1, which is applicable for classical metal-graphene transistor or a metal-graphene transistor on a silicon substrate. The device architecture presented in ref. 7 could be equivalent to that in Fig. 1, when the gate-oxide-semiconductor structure can be treated as a parallel-plate capacitor (it implies that the electric field at the graphene/silicon interface perpendicular to the interface *E*_G_ equals to zero in Fig. 1). The channel in such a top-gated GFET (Fig. 1) is divided into the source region and the drain region. It is assumed that the electrons are widely spread over the graphene in the drain region and the channel electron, enclosed by the rectangle ABCD in Fig. 1, have the Gauss’s surface. The saturation point is defined as *x* = 0, and the electric field outside the graphene layer is set as zero due to the screen effect by channel electrons. In other words, such a structure shown in Fig. 1 can be treated as a parallel-plate capacitor (the gate and the graphene can be regarded as parallel-plate terminals). The total of charge density (the charge density in the gate plus the charge density in the graphene) should be zero, therefore the electric field in the outside region of such a parallel-plate capacitor is zero according to the Gauss law, which is denoted as *E*_G_ = 0. Similar to the surface potential method for metal-oxide-semiconductor field effect transistor (MOSFET)^15^, the Gauss law is applied to the sides of the rectangle ABCD shown in Fig. 1 under saturation bias conditions,


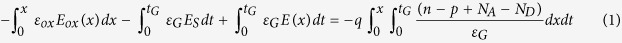


where 
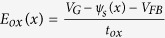
, 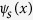 is the surface potential at *x, x* is the coordinate along the channel (as shown in Fig. 1), *V*_G_ is the gate voltage, *V*_FB_ is the flat band voltage, *t*_G_ is the thickness of the graphene layer, *t*_ox_ is the thickness of the gate oxide, *q* is the electron charge, ε_G_ is the dielectric constant of the graphene, ε_ox_ is the dielectric constant of the gate oxide, 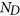
and 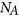 are the donor and acceptor concentrations, respectively, *n* is the electron density, *p* is the hole densities. The surface potential in the channel can be expressed as^16^:


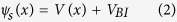


where *V*(*x*) is the channel surface potential at *x*, 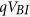 is the built-in potential energy from Fermi level of the source to the Fermi Level of the channel. Similar to the method in^16^, for *p*-type graphene, one obtains


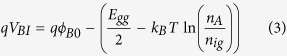


where *E*_gg_ is the band gap of the graphene and *E*_gg_ = 0 for monolayer graphene, *qϕ*_*B*0_ is the SBH (initial barrier height without lowering), *n*_A_ is the donor doping areal density in the graphene layer, *k*_B_ is the Boltzmann constant, *T* is the temperature, and *n*_ig_ is the intrinsic carrier areal concentration of graphene. Thus the derivation of Eq. 1 for *p*-type graphene under depletion approximation can be written as^16^:


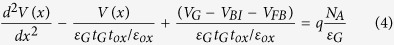


Using the boundary conditions *V*(0) = *V*_sat_ (*V*_sat_, the saturation voltage at onset of the saturation region, the position of *x* = 0 is the separating point between the source and drain regions), *V*(Δ*L*) = *V*_D_ (V_D_ is the drain voltage, Δ*L* is the length of the saturation region), *E*(0) = *E*_sat_ (*E*_sat_ is the saturation channel electric field)^15^, Eq. 4 can be solved to obtain V(x) as,


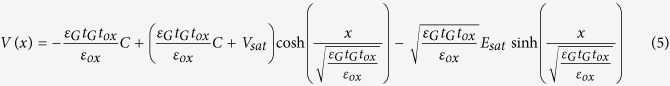


where 
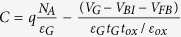
. Thus the channel electric field distribution can be determined as:


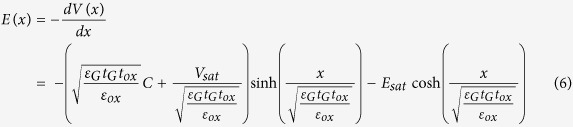


In order to determine the effective channel length *L*_E_, the length of the saturation region Δ*L* is required, the channel potential can be solved at *x* = Δ*L,*


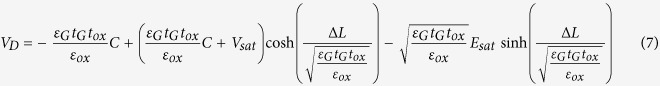


Note that the lateral electric field along the channel in the source region can be treated as the gradual channel approximation^17^ and the source voltage is 0 V, thus 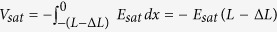 (here *L* is the channel length), Substituting 
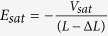
 into the Eq. 7, and one obtains


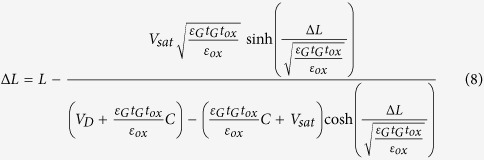


For simplicity, the lateral electric field in the drain region along the channel can be treated to be linear^15^


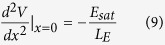


where *L*_E_ = *L* – Δ*L* is the effective channel length. Solving Eq.1 with the boundary condition (Eq. 9), one obtains


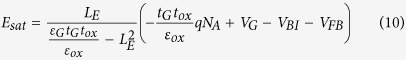


Thus the following is obtained:


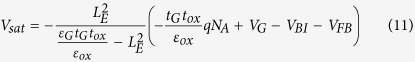


Assuming that 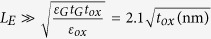 (for example *t*_ox_ = 100 nm, 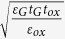 = 21 nm, *t*_ox_ = 1nm, 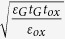 = 2.1 nm, thus it is found that, for realistic GFETs, such a condition is satisfied in most cases. Eq. 10 and Eq. 11 can be simplified as


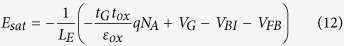



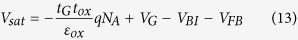


Furthermore, assuming that 
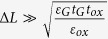
, thus one obtains that 
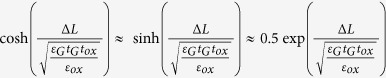
. Therefore, Eqs 5, 6, and 8 can be further simplified as


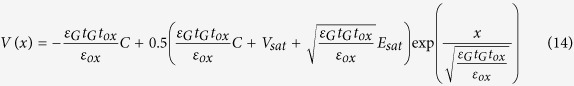



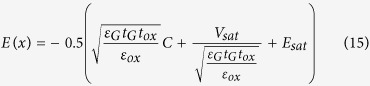



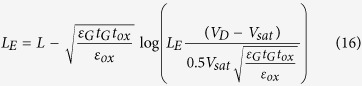


In a field-effect transistor, a lateral electric field in the channel not only results in a drift motion of electrons in the channel, but also changes their disordered thermal motion (the electron energy or electron temperature). Thus, the correlation between the electron temperature and lattice temperature in the channel of a transistor under the saturation bias conditions is given as^16^


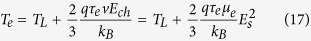


where *μ*_e_ is the mobility of electrons, *τ*_e_ is the ER time of electrons, *v* is the electron velocity, *T*_L_ is the lattice temperature (device temperature), and *E*_ch_ is the lateral electric field along the channel. Based on the gradual channel approximation, the effective SBH seen by the channel electrons when they obtain energy from the ER process can be written as


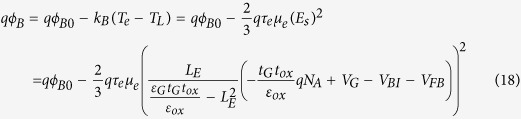


where *qϕ*_*B*_ is the effective SBH. Eq. 18 clearly shows that the effective Schottky barrier height will be affected by the difference between the electron temperature and lattice temperature. Larger difference between the electron temperature and lattice temperature could cause the larger reduction in the Schottky barrier height. According to the electron energy relationship, electron temperature increases with the square of the lateral electric field, electron energy relaxation time, and electron mobility. A lateral electric field in the channel can be analytically determined according to Eqns. 1–16. Because 
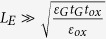
 is usually satisfied, Eq. 18 can be simplified as


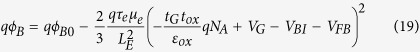


On the other hand, for *n*-type graphene, we just need to replace all (*N*_A_) with (-*N*_D_) in above equations. Then the similar equations for *n*-type graphene can be obtained.

For the source-drain current, the following equation of Schottky diode current-voltage relationship^16^ can be used


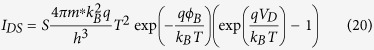


where *S* is the contact area between the source (drain) electrode and the channel (graphene), *h* is the Planck constant, and m^*^ is the effective mass of graphene. Therefore the source-drain current with consideration of the channel electrons ER can be written as


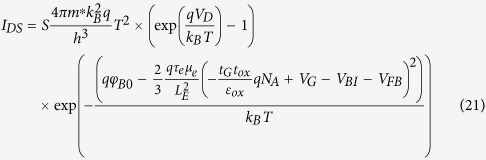


## Discussion

In our simulation, main parameters are given as following. The dielectric constant of 2.4 is used for graphene^18^. The thickness of single layer graphene is 0.34 nm^19^. According to other reports that the work function of graphene is considered as 4.5 eV in^20^ and 4.4 eV in^21^, the work function of graphene is considered as 4.5 eV in this paper^21^. The band gap of 1.12 eV is used for Si^16^. The relative dielectric constant of SiO_2_, Si, and the published electron affinity of SiO_2_ and Si are 3.9, 11.9, 0.9 eV and 4.05 eV, respectively [e.g.^22^,^23^, and references therein]. The ER time is 1 ps^2^, and the mobility is in the range of 1000–200000 cm^2^ V^−1^ s^−1^ [3–5, the supplementary materials of 7]. International units have been used in all calculations.

It was experimentally reported that the effective SBH between graphene and silicon in a graphene device strongly depends on the gate voltage^7^. Kim, T. G. *et al* thought that such a SBH lowering effect should originate from the image potential in silicon nanowire field effect transistors (FET), where as the experimentally extracted SBH of 0.5 eV is smaller than that of 0.55 eV from the simulation^8^, and the effective SBH could be expressed as^8^,^24^,


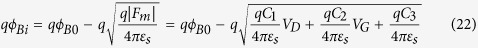


where *t*_s_ is the thickness of the nanowire, ε_s_ is its dielectric constant of the graphene, *qϕ*_*B*i_ is the effective SBH with consideration of the image force, the *C*_1_, *C*_2_, and *C*_3_ are fitting parameters. And the channel surface electric field is^24^


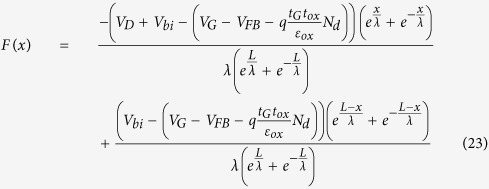


where 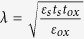. Thus^24^


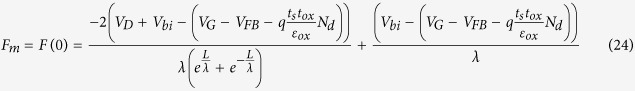


and


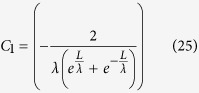



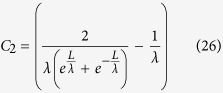


Using the parameters in^24^, for example 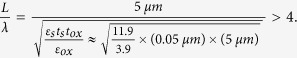 Therefore, 
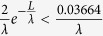
 and 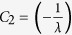 because 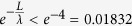, and


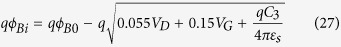


*C*_1_, *C*_2_, and *C*_3_ are just fitting parameters in the former studies^8^,^24^. Solving Eqns. 25, 26, 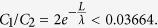 However, extracted from experimental results, 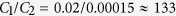 for the positive *V*_D_, significant larger that the above theoretically calculation value, which denotes that the image force can not give a good explanation on the experimental data of the gate controlled SBH.

Therefore, the hot-electron effect is adopted to theoretically investigate the gate controlled SBH in graphene. In the following results, the different effects of the image potential and the ER of channel electrons on the SBH lowering are shown, and the comparison of simulation results with experimental results of silicon nano-wire FETs is given.

**Figure 2:**
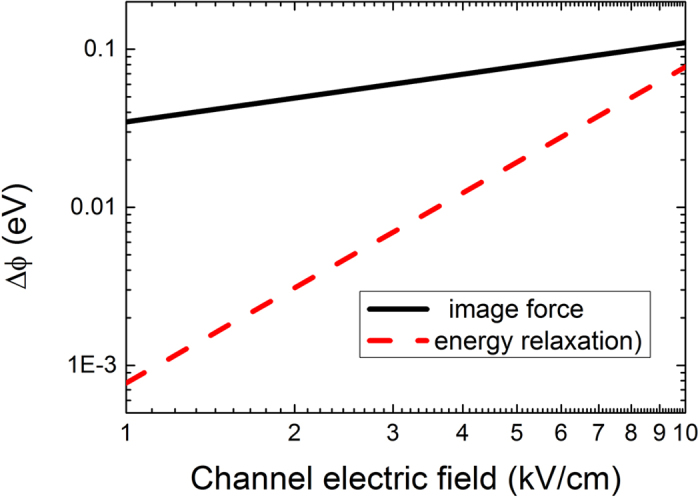


Figure 2 shows the reduction in the SBH (or the SBH lowering), 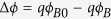, caused by the image force (Eq. 22) and the ER ((Eq. 18)), respectively, as a function of the lateral channel electric field. In Fig. 2, the ER time of 0.8 ps at 300 K^26^ and the mobility of 1450 cm/V·s^16^ for electrons in silicon have been used. For *V*_G_ = 0, it could be found that the 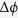 between *V*_D_ = 0 V and *V*_D_ = 1 V is around 0.02 eV according to the experimental data from Fig. 7 of ref. 24. For simplicity, the lateral channel electric field could be simplified as 
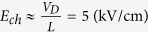
 at *V*_D_ = 1 V and *V*_G_ = 0. It is easily found in Fig. 2 that the reduction of the SBH 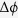 caused by the image force is around 0.078 eV at the channel electric field of 5 kV/cm, whereas the 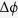 caused by the ER of channel electrons is around 0.019 eV. Fig. 2 proves the validity of the ER of channel electrons as the physical mechanism of the SBH lowering in field-effect transistor by comparing the difference of SBH lowering induced by the image force and ER of channel electrons with experimental results.

According to Eq.22, the SBH lowering (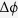) caused by the image force is proportional to the square root of the gate voltage, which denotes that 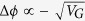 or 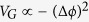. While the SBH lowering caused by the ER of electrons is proportional to the square of the gate voltage according to Eq. 18, which denotes that 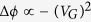 or 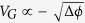. The further comparison of the reductions in SBH calculated by the image potential (the ER of channel electrons) and extracted from experimental data in ref. 7 is given as following.

**Figure 3:**
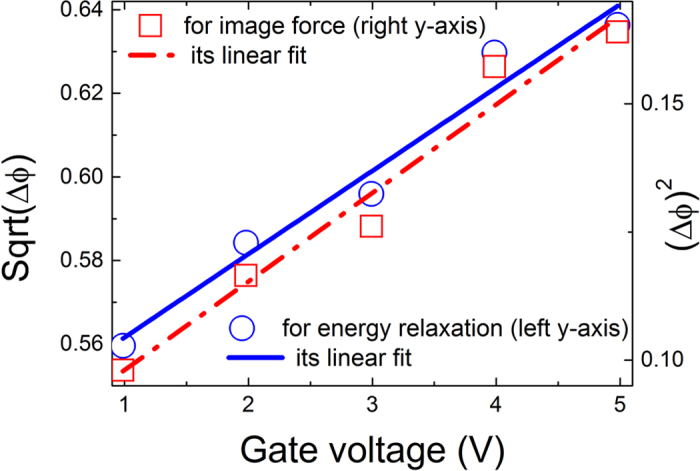
The experimental values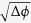 and 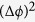 as a function of the gate voltage, experimental data from ref. 7.

Figure 3 shows that the square of the SBH reduction 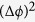 and the square root of the SBH reduction (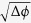) as a function of the gate voltage (the symbols in Fig. 3 are the experimental data that come from Fig. 3 B in ref. [7], and the SBH value of 0.67 eV is used to estimate 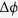). Although both methods for the SBH lowering using the image force and the ER of channel electrons could be used to explain the SBH lowering observed in the experimental results, which is shown in Fig. 3. Experimental extracted 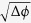 ~ Vg has a slightly better linear fitting in comparison with the linearly fitting of 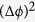 ~ Vg. The adjusted R-Square is 0.95557 for linearly fitting of 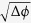 to *V*_G_, and the adjusted R-Square is 0.95308 for linearly fitting of 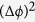 to *V*_G_, which demonstrates that the SBH lowering caused by the ER of channel electrons agrees better with the experimental results.

For the SBH lowering in the graphene/*p*-Si contacts, the maximum field graphene/*p*-Si contact under the abrupt approximation can be written as^16^


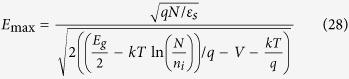


where *E*_g_ = 1.12 eV is the band gap of silicon. For a Schottky junction, the reduction in SBH caused by the ER of electrons is 
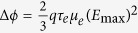
 according to Eq.18 (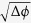 should be linearly dependent on *E*_max_), whereas the SBH caused by the image force is 
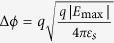
 according to Eq. 22 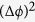 should be linearly dependent on *E*_max_).

**Figure 4:**
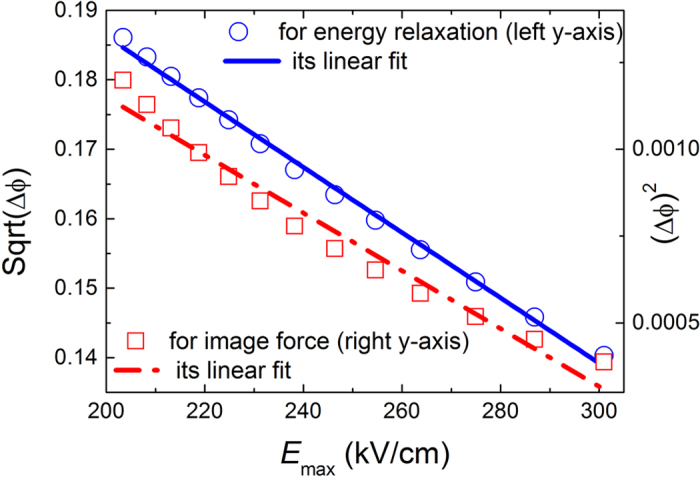
The experimental values 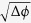 and 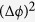 as a function of the maximum electric field in the graphene/p-Si contact, according to ref. 27.

Figure 4 shows how experimental values of the 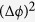
and 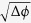 change with the maximum electric field in the graphene/p-Si contact (the symbols in Fig. 4 are the experimental data and come from Fig. 2 C of ref. [27]). In Fig. 4, it is also clearly shown that there is a better linear fit between experimental extracted 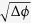 and *E*_max_ in comparison with the curve of 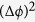 and *E*_max_. The adjusted R-Square is 0.99584 when we linearly fit *E*_max_ to 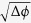, and the adjusted R-Square is 0.96622 when we linearly fit *E*_max_ to 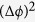. It indicates again that the SBH lowering caused by the ER is more in accord with the experimental results. All above results show that modeling the SBH lowering by using the ER of channel electrons in semiconductor devices is consistent with experimental results.

In the following, we will discuss the detailed influence of the ER of channel electrons on the gate controlled SBH lowering. It should be noted that the saturation density of *n*-type doping in monolayer graphene is around 1 × 10^13^ cm^−2^ 28 and the net *p*-type doping in bilayer and monolayer graphene is around 2 × 10^13^ cm^−2^ 29. It was reported in^30^ that the saturation characteristic of top-gated GFET degrades with the channel length shrinking from 5.6 μm down to 100 nm, and complete saturation can occur at the channel length of 5.6 μm. The source and drain material of *p*-type silicon is used in the following calculations. It should be noted that the electron velocity in a graphene can reach 1 × 10^6^ m/s^1^, thus the distance of electron transport in the graphene during 1 ps is 1 μm. For the energy balance conditions, all channel length used in the following calculations is larger than 1 μm. And *V*_FB_ = 0 is chosen in the all calculations of this work.

**Figure 5:**
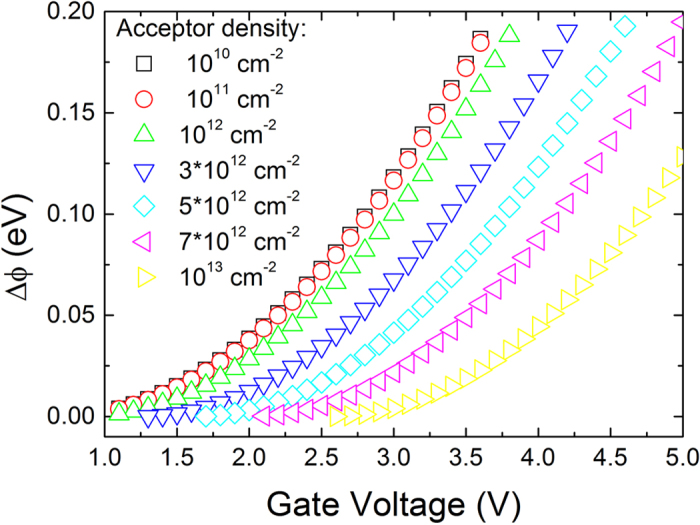
The reduction of the SBH as a function of the gate voltage with the HfO_2_ layer thickness of 20 nm for different acceptor density. The channel length is 2 μm, the drain voltage is 3 V, the electronic mobility is 1300 cm^2^ V^−1^ s^−1^, the ER time is 1 ps and the device temperature is 300 K.

Figure 5 shows how the reduction of the SBH caused by the relaxation of channel electrons as a function of the gate voltage for different acceptor densities, where for larger acceptor doping in a GFET a larger gate controlled SBH lowering could be observed. From Fig. 5, it can be concluded that the effective SBH between graphene and electrode in a GFET decreases with the increasing of gate voltage, which clearly shows that the SBH lowering in a GFET can be modulated by applying different gate voltages due to the ER of channel electrons.

**Figure 6:**
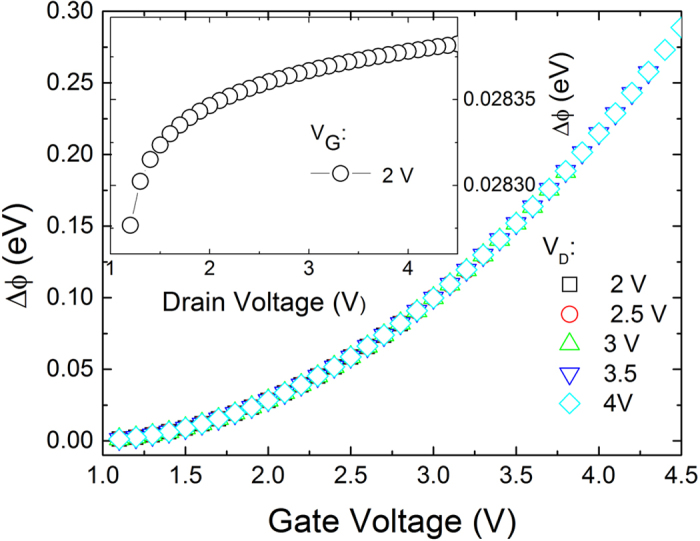
The reduction of the SBH as a function of the gate voltage with the HfO_2_ layer thickness of 20 nm for different drain voltage. The inset figure shows the reduction of the SBH as a function of drain voltage when the gate voltage is 2 V. The channel length is 2 μm, the drain voltage is 3 V, the acceptor density in graphene is 1 × 10^12^ cm^−2^, the electronic mobility is 1300 cm^2^ V^−1^ s^−1^, the ER time is 1 ps and the device temperature is 300 K.

Figure 6 shows how the reduction of the SBH caused by ER of electrons changes with the gate voltage for different drain voltages. The inset of Fig. 6 shows that the reduction in the SBH caused by the ER of channel electrons slightly increases with the drain voltage. Because the saturation voltage is independent on the drain voltage according to Eq.13 and the relative small change in the effective channel length is caused by the drain voltage according to Eq. 16, the drain voltage could hardly reduce the SBH according to Eqs. 18 or 19.

**Figure 7:**
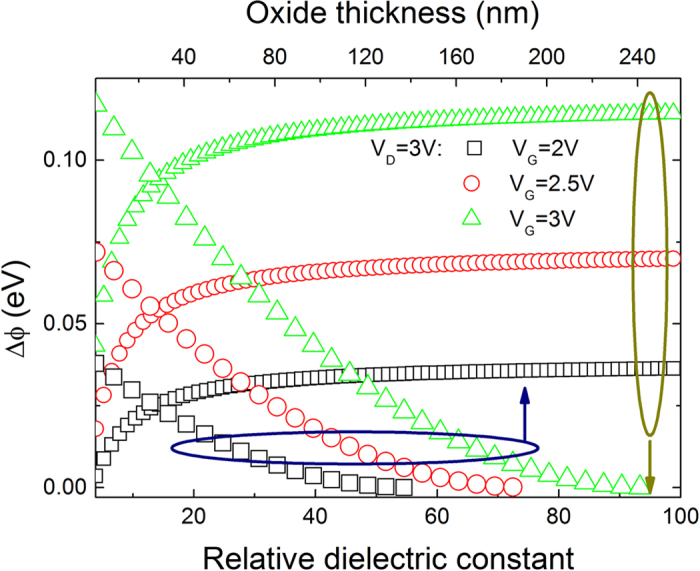
The reduction of the SBH as a function of the relative dielectric constant of the gate oxide with 20 nm thickness. The channel length is 2 μm, the acceptor density in graphene is 1 × 10^12^ cm^−2^ the electronic mobility is 1300 cm^2^ V^−1^ s^−1^, the ER time is 1 ps and the device temperature is 300 K.

Figure 7 shows that the reduction of the SBH caused by the ER of channel electrons as function of the relative dielectric constant and thickness of the gate oxide in a GFET. It is observed that the reduction of the SBH rapidly increases firstly with the relative dielectric constant of the gate oxide in a GFET, then approaches to a saturation value for the large relative dielectric constant (>20), which implies that gate insulator materials with high relative dielectric constant (>20) should be chosen for the best gate controlled SBH lowering effects in a GFET. Fig. 7 also shows the reduction of the SBH decreases with a thicker oxide since the saturation voltage and electric field decreases with the increasing of oxide thickness according to Eq. 13 and Eq. 12.

**Figure 8:**
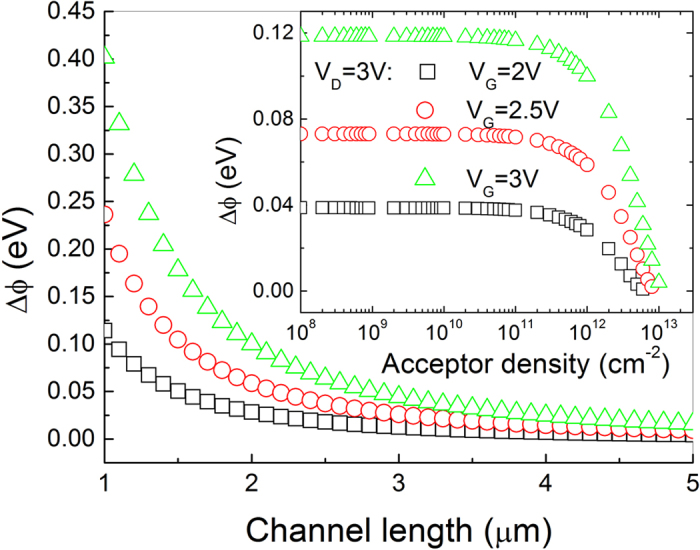
The reduction of the SBH as a function of the channel length when the acceptor density in graphene is 1 × 10^12^ cm^−2^. The inset figure shows the reduction of the SBH in as a function of the acceptor density when the channel length is 2 μm. The HfO_2_ layer thickness is 20 nm, the electronic mobility is 1300 cm^2^ V^−1^ s^−1^, the ER time is 1 ps and the device temperature is 300 K.

Figure 8 shows how the reduction of the SBH between the graphene and electrode in a GFET changes with the channel length for different gate voltages under a given drain voltage. The reduction in the SBH firstly rapidly decreases with increasing channel length, then saturates with further increasing of channel length. It is because the strength of saturation electric field along the channel is the reciprocal of the effective channel length according to Eq. 12, under the approximation assumption of gradual channel. It should be noted that the reduction of the SBH is a reciprocal function of the square of the effective channel length according to Eq. 18 or Eq. 19, thus the reduction of the SBH rapidly deceases with the increasing of channel length. Such a reduction is too small to be observed when the channel length is large enough. The inset figure in Fig. 8 illustrates that a change in doping density for small acceptor doping in graphene (<1 × 10^11^ cm^−2^) have almost no effect on the effective SBH, but a change in doping density for larger acceptor doping in graphene (>1 × 10^11^ cm^−2^) could significantly affect the effective SBH.

Though the models presented in the previous literature work^31^,^32^ could describe the current-voltage characteristics of GFETs, it is difficult to analytically formulate Schottky barrier height lowering effects. In these models, many parameters have been used for Schottky barrier height lowering effect; where some fitting parameters have to be adopted to keep consistent with experimental results. Therefore, the lack of concise expression for the gate-controlled Schottky barrier height lowering effects limits the practical application of these methods. In our proposed model, main parameters still provide clear physical meanings, especially for the Schottky barrier height lowering effect, indicating that these model parameters could be expressed by device parameters, such as relative dielectric constant, channel density, doping density, and the energy relation time, which ensure device optimization design of the high-performance graphene field effect transistors. At the same time, due to general validity of electron transport of Eqs. 17 and 18 in various semiconductor materials, the proposed model could be extended to non-graphene materials and other device architectures only if the channel electric field is known. In others words, we can obtain similar conclusions by using the derivation of the channel electric field in other device architectures.

## Conclusions

In conclusion, the effect of the ER (Energy Relaxation) of channel electrons on the SBH between graphene and electrode in a GFET has been theoretically investigated and physically modeled. The theoretical calculations agree well with experimental data reported in ref. [7,^24^,^27^]. The ER of channel electrons can result in a high electron temperature, thus causing a larger reduction in the SBH between graphene and electrode. Based on the energy conservation equation with the balance assumption, a physical model is built in this work to describe the gate controlled SBH lowering effects in a GFET under the saturation mode. The increases in the electron mobility and the ER time of channel electron will result in a linear increase in the reduction in the SBH according to the proposed model (Eq. 19). And the effects of parameters such as oxide thickness, oxide relative dielectric constant, channel length, drain voltage, and acceptor density are analyzed in detail. The drain voltage has slightly effect on the reduction of the SBH. The increase of the gate oxide thickness, the acceptor density in the graphene, and the channel length result in the decrease of SBH reduction magnitude. Whereas, increase in the dielectric constant of the gate oxide and the gate voltage result in obvious SBH reduction. All these results indicate that the effect of electron ER on the reduction in SBH should be seriously taken into account in GFETs with nano-scale dimension.

## Additional Information

**How to cite this article**: Mao, L.-F. *et al.* Physical Modeling of Gate-Controlled Schottky Barrier Lowering of Metal-Graphene Contacts in Top-Gated Graphene Field-Effect Transistors. *Sci. Rep.* **5**, 18307; doi: 10.1038/srep18307 (2015).

## References

[b1] Novoselov K. S. *et al.* Two-dimensional gas of massless Dirac fermions in graphene. Nature 438, 197–200 (2005). 16281030 10.1038/nature04233

[b2] Tiras E. *et al.* Effective mass of electron in monolayer graphene: Electron-phonon interaction. J. Appl. Phys. X 113, 043708-1-7 (2013).

[b3] Verma R., Bhattacharya S. & Mahapatra S. Modeling of temperature and field-dependent electron mobility in a single-layer graphene sheet. IEEE Trans. Electron Dev. 60, 2695–2698 (2013).

[b4] Kotin I. A. *et al.* High carrier mobility in chemically modified graphene on an atomically flat high-resistive substrate. J. Phys. D: Appl. Phys. 46, 285303-1-6 (2013).

[b5] Zwierzycki M. Transport properties of rippled graphene. J. Phys.: Condens. Mat. 26, 135303-1-9 (2014). 10.1088/0953-8984/26/13/135303 24614231

[b6] Tongay S., Schumann T., Miao X., Appleton B. R. & Hebard A. F. Tuning Schottky diodes at the many-layer-graphene/semiconductor interface by doping. Carbon 49, 2033–2038 (2011).

[b7] Yang H. *et al.* Graphene barristor, a triode device with a gate-controlled Schottky barrier. Science 336, 1140–1143 (2012) 22604723 10.1126/science.1220527

[b8] Kim T. G. *et al.* Barrier Height at the Graphene and Carbon Nanotube Junction. IEEE T. Electron. Dev. 61, 2203–2207 (2014).

[b9] Hatakeyama T., Ishizuka M., Nakagawa S. & Fushinobu K. Electro-Thermal Analysis and Monte Carlo Simulation for Thermal Design of Si Devices. Trans. Jpn. Inst. Electron. Packag. 4, 61–67 (2011).

[b10] Fushinobu K. & Hatakeyama T. Electro-thermal scaling analysis of Si MOSFETs with device length typically larger than 100 nm. Trans. Jpn. Inst. Electron. Packag. 4, 31–35 (2011).

[b11] Rao H. & Bosman G. Hot-electron induced defect generation in AlGaN/GaN high electron mobility transistors. Solid State Electron. 79, 11–13 (2013).

[b12] Eless V. *et al.* Phase coherence and energy relaxation in epitaxial graphene under microwave radiation. Appl. Phys. Lett. 103, 093103-1-3 (2013).

[b13] Sun D. *et al.* Ultrafast relaxation of excited Dirac fermions in epitaxial graphene using optical differential transmission spectroscopy. Phys. Rev. Lett. 101, 157402-1-4 (2008). 10.1103/PhysRevLett.101.157402 18999638

[b14] Sun D. *et al.* Ultrafast hot-carrier-dominated photocurrent in graphene. Nat. Nanotechnol. 7, 114–118 (2012). 22245859 10.1038/nnano.2011.243

[b15] El Banna M. & El Nokali M. A pseudo-two-dimensional analysis of short channel MOSFETs. Solid State Electron. 31, 269–274 (1988).

[b16] Sze S. M. & Ng K. K. Physics of semiconductor devices. 3rd edition, (John Wliey & Sons, New York, 2007).

[b17] Terrill K. W., Hu C. & Ko P. K. An analytical model for the channel electric field in MOSFETs with graded-drain structures. IEEE Electron Dev. Lett. 5, 440–442 (1984).

[b18] Lemme M. C., Echtermeyer T. J., Baus M. & Kurz H. A Graphene Field-Effect Device. IEEE Electron Dev. Lett. 28, 282–4 (2007).

[b19] Schmidt H. *et al.* Tunable graphene system with two decoupled monolayers. Appl. Phys. Lett. 93, 172108-1-3 (2008).

[b20] Yu Y. J. *et al.* Tuning the Graphene Work Function by Electric Field Effect. Nano Lett. 9, 3430–3434 (2009). 19719145 10.1021/nl901572a

[b21] Giovannetti G. *et al.* Doping graphene with metal contacts. Phys. Rev. Lett. ; 101, 026803-1-4 (2008). 10.1103/PhysRevLett.101.026803 18764212

[b22] Mao L. F., Zhu C., Zhang L., Ji A. & Liu X. Investigation of Crystal Size Impacts on the Tunneling Current in Germanium Nanocrystal Metal-Oxide-Semiconductor Transistors. Curr. Nanosci. 9, 103–106 (2013)

[b23] Mao L. F. Quantum coupling effects on charging dynamics of nanocrystalline memory devices. Microelectron. Reliab. 54, 404–409 (2014).

[b24] Lee S. H., Yu Y. S., Hwang S. & Ahn D. A SPICE-compatible new silicon nanowire field-effect transistors (SNWFETs) model. IEEE T. Nanotechnol. 8, 643–649 (2009).

[b25] Chang W., Shih C. H., Luo Y. X., Wu W. F. & Lien C. Drain-induced Schottky barrier source-side hot carriers and its application to program local bits of nanowire charge-trapping memories. Jpn. J. Appl. Phys. 53, 094001-1-5 (2014).

[b26] Brugger S. C. & Schenk A. First-principle computation of relaxation times in semiconductors for low and high electric fields. Proceedings of the International conf. on Simulation of Semiconductor Processes and Devices, Piscataway NJ (USA), 151–154 (2005).

[b27] An Y., Behnam A., Pop E. & Ural A. Metal-semiconductor-metal photodetectors based on graphene/p-type silicon Schottky junctions. Appl. Phys. Lett. 102, 013110-1-5 (2013).

[b28] Kopylov S., Tzalenchuk A., Kubatkin S. & Fal’ko V. I. Charge transfer between epitaxial graphene and silicon carbide. Appl. Phys. Lett. 97, 112109-1-3 (2010).

[b29] Zhou S. Y., Siegel D. A., Fedorov A. V. & Lanzara A. Metal to insulator transition in epitaxial graphene induced by molecular doping. Phys. Rev. Lett. 101, 086402-1-4 (2008). 10.1103/PhysRevLett.101.086402 18764644

[b30] Bai J. *et al.* Top-gated chemical vapor deposition grown graphene transistors with current saturation. Nano Lett. 11, 2555–2559 (2011). 21548551 10.1021/nl201331x PMC3236244

[b31] Jimenez D. & Moldovan O. Explicit drain-current model of graphene field-effect transistors targeting analog and radio-frequency applications. IEEE T. Electron. Dev. 58, 4049–4052 (2011).

[b32] Pugnaghi C. *et al.* Semianalytical quantum model for graphene field-effect transistors. J. Appl. Phys. 116, 114505 (2014).

